# Cognitive impairment and driving: A review of the literature

**DOI:** 10.1590/S1980-57642009DN30400004

**Published:** 2009

**Authors:** Daniel Apolinario, Regina Miksian Magaldi, Alexandre Leopold Busse, Leonardo da Costa Lopes, Juliana Yumi Tison Kasai, Erika Satomi

**Affiliations:** 1,4,5,6 MD, Memory and Aging Unit, Geriatric Service, Department of Clinical Medicine, University of São Paulo School of Medicine, São Paulo SP, Brazil.; 2 MD, Assistant Physician, Memory and Aging Unit, Geriatric Service, Department of Clinical Medicine, University of São Paulo School of Medicine, São Paulo SP, Brazil.; 3 MD, PhD, Memory and Aging Unit, Assistant Physician, Geriatric Service, Department of Clinical Medicine, University of São Paulo School of Medicine, São Paulo SP, Brazil.

**Keywords:** automobile driving, cognition, dementia, aged

## Abstract

Although some drivers with mild dementia may continue to drive after the condition has been diagnosed, the ability to drive a motor vehicle safely is eventually lost as the disease progresses. Clinicians involved in dementia care are often asked to make an assessment on whether a patient is fit to drive, even though they often lack basic knowledge and formal training in this area. The purpose of this review was to identify the factors that may differentiate safe from unsafe drivers with cognitive impairment and to discuss management strategies. Isolated information about staging measures or particular cognitive tests was found to be insufficient for decision making. Driving fitness counseling for patients with cognitive impairment requires a solid knowledge base, comprehensive assessment and thoughtful communication.

## Abstract (pt)

Alguns pacientes com demência leve continuam a dirigir após o diagnóstico, mas a habilidade de conduzir um veículo automotor com segurança é perdida à medida que a doença progride. Apesar de muitas vezes não reunirem conhecimento e treinamento formal para essa tarefa, os profissionais de saúde envolvidos na atenção ao paciente com demência precisam emitir recomendações relativas à capacidade desses indivíduos em conduzir um veículo. Essa revisão tem o objetivo de identificar fatores que possam determinar se um indivíduo com comprometimento cognitivo é capaz de conduzir um veículo com segurança. Informações isoladas relativas ao estadiamento ou a testes cognitivos específicos não parecem ser suficientes para uma tomada de decisão apropriada. O aconselhamento nessa situação exige conhecimentos sólidos aplicados a uma avaliação ampla, além de estratégias de comunicação apropriadas ao contexto.

Driving is a complex task that involves a wide range of cognitive skills, multisensory perception, and motor abilities. Cognitive impairment, in its varying degrees, has been identified as an important factor that can affect the ability of older adults to drive a motor vehicle.^1^ As populations age and the proportion of older drivers on the road rises, health care professionals will increasingly become involved in decisions about driving by individuals with cognitive impairment.^2,3^

In the past, some authors suggested that a diagnosis of dementia indicated the need to stop driving immediately,^4^ but this oversimplification becomes unacceptable as we move towards the concepts of person-centered care, which attempts to preserve mobility and independence for as long as possible after the onset of dementia.^5,6^ There is now sufficient evidence to suggest that not all persons with dementia are hazardous drivers, and that some of these individuals may continue to drive if they are regularly reevaluated.^7,8^ It is important, however, to underscore that drivers with progressive dementia will eventually need to stop driving. The issue in this case is not a question of “if” stopping driving will be necessary, but “when”.

In clear-cut cases such as moderate to severe dementia, prohibitive risks are a matter of common sense, but making recommendations in milder cases may be a more challenging task. There is lack of consensus on how to determine whether a driver with cognitive impairment is still able to operate a motor vehicle safely. Valid and simple instruments for assessing driving fitness in clinical practice are also lacking. Specialized on-road tests are expensive and largely unavailable in most countries. In this context, a broad knowledge base is essential for the health care professional when trying to strike an adequate balance between personal autonomy and public safety. ^9^

In this article we report the results of an integrative review to describe the impact of cognitive impairment on driving abilities and to address the yield of screening tools that have been proposed for identifying hazardous driving. Finally, management strategies are discussed with the aim of guiding health care providers in evaluating and counseling for driving safety.

In preparing for this review, we carried out a literature search from 1999 to September 2009 of several electronic bases (Medline, PubMed, ScieLO, LILACS, and the Cochrane Library). Keywords used individually and in various combinations included: driving, cognitive, dementia, Alzheimer’s disease, and accidents. The references generated were checked for relevance on the basis of their title and abstract, and we followed up other references from the papers identified.

## Epidemiology

Studies in different populations have assessed the prevalence of dementia in older drivers and, from the opposite perspective, the proportion of individuals diagnosed with dementia who still drive. In a study conducted in the American state of North Carolina, cognitive impairment was detected in 20% of the drivers aged 80 and older who attempted to renew their licenses.^10^ In the Honolulu-Asia Aging Study (HAAS), 30% of the participants diagnosed with dementia were driving. Based on these data, it was estimated that in United States 4.4% of the male drivers aged 75 years and older have dementia.^11^

Longitudinal studies have found that individuals with very mild to mild dementia continue to drive for a surprisingly long period of time. Herrmann et al. followed 203 demented drivers from the Canadian Outcomes Study in Dementia (COSID) who had an average age of 74.5 years and a mean Mini-Mental State Examination (MMSE) score of 24.25 at baseline. In this sample, 51.5% were still driving 2 years later.^12^ Other cohort studies have found similar results, indicating that about half the drivers diagnosed with dementia still drive after a period of 2 to 3 years.^11,13^

In older subjects diagnosed with cognitive impairment, a number of factors have been identified that influence driving behavior and the decision to stop driving. When analyzing socio-demographic factors, older age has been shown in virtually all cohorts as a strong predictor for earlier driving cessation.^13-17^ In a less consistent way, lower educational attainment^12^ and female gender^16,17^ were associated with a greater probability of driving cessation in some studies.

Lower baseline scores on global cognitive measures such as the MMSE have been found to predict an increased likelihood of driving cessation.^12^ In a prospective study that included 53 older men with dementia, lower MMSE scores and older age were the only predictive factors for driving cessation.^13^

As would be expected, classification into more advanced dementia stages appears to be a strong risk factor for driving cessation. The HAAS study consisted of a retrospective cohort of a total of 643 men with a mean age of 80.6 years who were evaluated for dementia. Driving exposure dropped sharply according to increased Clinical Dementia Rating (CDR) stage. Among men with normal cognitive functioning (CDR 0), 78% still drove, compared with 46% of men with very mild dementia (CDR 0.5), and 22% of men with mild dementia (CDR 1). Only one out of 23 men diagnosed with moderate dementia (CDR 2) was driving.^11^

In a 3-year prospective study that assessed several clinical, functional and cognitive measures in individuals with dementia, the stage of Global Deterioration Scale (GDS), a measure of severity, was found to be the main factor associated with eventual driving cessation. It is noteworthy that in this study the presence of neuropsychiatric symptoms significantly increased the probability of quitting driving. Introduction of the individual Neuropsychiatric Inventory (NPI) items into the model revealed that the presence of apathy or hallucinations were predictive of driving cessation, whereas agitation was associated with a reduced risk of stopping driving.^12^

Studies associating driving cessation to living arrangements have shown that household characteristics play an important role in moderating the association between cognitive functioning and driving. Data from a sample of 5460 community-dwelling adults aged 70 or older from the Asset and Health Dynamics Among the Oldest Old (AHEAD) showed that cognitively impaired individuals with alternate drivers in the household are more likely not to drive.^18^ Thus, having other drivers in the household seem to encourage earlier driving cessation. In a retrospective study of 430 consecutive patients referred to a memory clinic, participants living outside cities were more likely to continue driving than those in cities, which could be explained by the lack of suitable transportation alternatives and the longer distances to be covered outside urban areas.^15^

Adler et al. used information obtained from collateral sources to study the factors that influence the decision to stop driving in individuals with dementia. Even in the presence of degenerative dementia, only 9.3% of the participants had made plans for driving cessation at baseline, although 46.5% had completed an advance directive. Fittingly, most collaterals reported that the decision to stop driving was abrupt (60.9%). When the decision to stop driving was made, physicians were identified by collaterals as the decision-maker in 56.5% of such cases.^13^

## Cognitive functioning and driving performance

The degree to which cognitive impairment affects driving performance and which cognitive domains best correlate with driving abilities have been the subject of extensive research in recent decades. Measures of driving performance are obtained basically through on-road tests or driving simulators. On-road tests are often considered the gold standard due to their high ecological validity, even though they are costly, liable to subjectivity in scoring, and incapable of controlling variables such as traffic flow, road conditions and other drivers’ behavior. More recently, interactive driving simulators have been developed which are realistic and provide an objective means of standardizing a number of variables, including the placing of drivers in potentially dangerous situations.

### Dementia and driving performance

A recent systematic review was conducted to determine whether persons with dementia are poorer drivers than cognitively normal drivers. A total of 19 studies provided data regarding driving performance, all using a case-control design. The results clearly demonstrated that persons with dementia did not perform as well as control subjects in tests of driving performance, including on-road and driving simulator evaluations. The most frequent safety errors committed by drivers with dementia were related to difficulties in maintaining lane control, driving too slowly, and taking excessive time to turn left at intersections.^19^

To assess driving performance in relation to dementia severity, Berndt et al. recruited 115 licensed drivers from a memory clinic in Australia. The patients were assessed using a standardized on-road test and had overall pass or fail outcomes determined by an occupational therapist blind to the severity of dementia. Drivers with moderate dementia generally failed the assessment, but in the very mild to mild dementia range the results were equivocal. Pass rates for drivers with CDR 0.5 and CDR 1 was 65% and 42%, respectively.^20^ These results suggest that the guideline by the Quality Standards Subcommittee of the American Academy of Neurology recommending that all CDR 1 individuals cease driving, may be too restrictive.^21^ In fact, for individuals with very mild or mild dementia, decision-making based exclusively on the severity level would promote either continued unsafe driving or premature cessation in an unacceptable number of cases.

### Mild Cognitive Impairment and driving performance

Recent studies have examined the driving performance of individuals with Mild Cognitive Impairment (MCI), as it has been hypothesized that driving may be added to the list of complex instrumental activities of daily living in which the performance of individuals with MCI is expected to be impaired. Wadley et al. compared 46 individuals with MCI (according to the Petersen criteria) to 59 cognitively normal controls on a driving evaluation. Participants with MCI were significantly more likely than controls to receive less than optimal ratings on left-hand turns, lane control, and overall rating. The adjusted OR indicated that MCI patients were 4.23 times more likely than controls to receive a less than optimal overall rating. Mean differences were small in magnitude and the performance decrements did not reach the level of significant impairment.^22^ Other studies have obtained similar results to those described above, which are consistent with the concept of MCI.^23,24^

### Neuropsychological domains and driving performance

Neuropsychological assessment may provide a noninvasive and relatively inexpensive way of predicting driving performance. A meta-analysis of 27 studies conducted by Reger et al. examined the relationship between specific cognitive domains and driving ability for subjects with dementia.^25^ As most neuropsychological tests assess multiple cognitive domains, each test was coded using a single domain that was judged to best represent the primary function assessed. To this end, tests were categorized into the following six neuropsychological domains: mental status (global cognition), attention, executive functions, language, visuospatial skills, and memory.

Visuospatial skills are required to achieve accurate positioning on the road, to judge distances and maneuver the automobile while predicting the development of traffic situations. Accordingly, in this meta-analysis, tests of visuospatial skills were the most strongly associated with driving abilities, with the magnitude of the effect being moderate for both on-road and non-road tests (r=0.29 and 0.31, respectively). In addition, the visuospatial tasks represented one of the few domains significantly correlated to the caregiver’s report of driving skills.

Several investigators have theorized that attention is an important skill to assess in drivers with dementia. However, correlation was not as strong as expected when all tests of attention were aggregated. The magnitude of the effect was not significant for non-road tests but significant, yet small (r=0.25), for on-road tests. These results may be attributed to the broad concept of attention adopted in this study. Identifying important information in the environment while ignoring irrelevant information may be an important driving skill. Thus, selective attention is thought to be more specific to driving deficits than other components such as divided and sustained attention. In future studies, it is possible that specific aspects of attention will be found to be associated with driving ability to a greater degree than the cognitive domain as a whole.

Executive function tests also showed relatively poor relationships to measures of driving ability. In view of these findings, it is important to note that tests with such different properties were selected to represent the domain. Trail Making B, Stroop, Mazes and Category Fluency tests were selected to represent executive functioning, although the Clock Drawing Test was considered a visuospatial skill. Correspondingly, the unexpected low correlations obtained may be ascribed to the loose concept of executive function and to the broad range of tasks used to represent the domain.

Mental status results in this meta-analysis were mixed. This pattern may reflect the fact that the mental status tests most frequently used, such as MMSE, have properties appropriate for the mild to moderate stages of dementia. Instead, cognitive measures adequate for studying driving abilities should have psychometric properties best suited for detecting small changes in the very mild to mild range of cognitive impairment.

The importance of overall cognitive function was recently demonstrated by Dawson et al, in a study that included 40 drivers with early Alzheimer disease (mean MMSE 26.5) and 115 drivers without dementia who underwent a standardized on-road evaluation.^26^ A composite cognitive score (COGSTAT) was calculated for each subject based on eight neuropsychological tests. After adjusting for age and gender, COGSTAT predicted safety errors in subjects with dementia better than any individual test. The authors concluded that driving places demand on diverse cognitive functions and it is unlikely that any single cognitive domain will be an accurate predictor of driving safety.

Dawson’s study also points out that memory impairment, generally considered to be the hallmark of Alzheimer disease (AD), is not a good indicator of driving ability. These findings agree with results reported in earlier studies which have found that even persons with severe amnesia can drive a motor vehicle without great difficulty.^27^

In a recent non-structured review, Silva MT et al. searched for specific neuropsychological measures that might be useful to predict driving competence of demented individuals. Tasks that assess executive processing, particularly those that also include visuomotor demands (e.g. Porteus Mazes, Trails B, Clock Drawing, Useful Field of View) were suggested to be useful to identify at-risk drivers with early dementia.^28^

Taken together, findings from studies relating driving performance to cognitive functions suggest that composite scores, visuospatial skills and perhaps executive functions should be considered in decisions about driving maintenance or cessation. Nevertheless, given the mere moderate correlations found in most studies, the decisions regarding fitness to drive should not be based exclusively on neuropsychological testing.

### Cognitive impairment and risk of accidents

Using straightforward reasoning, one might assume that cognitively impaired individuals run a greater risk of causing automobile accidents, as they perform poorly on driving abilities tests. However, in response to observed difficulties, drivers with dementia may restrict their driving to favorable circumstances and may have limitations placed by caregivers. Accordingly, the correlation between driving abilities and the risk of automobile accidents may be attenuated by adaptive behavior and compensatory strategies.

Three studies compared caregiver-reported crashes in patients with dementia and age-matched controls. In all three studies, drivers with dementia crashed more often than controls, with dementia conferring a 2.5 to 8-fold greater risk.^29-31^ However, studies using state driving records did not consistently find the same result. Of the three studies that used state crash records as the outcome measure, only one found higher rates of crashes in patients with dementia.^32-34^ Besides the compensatory strategies cited above, it may be the case that collision rates on state records are relatively infrequent events, thus rendering them an insensitive measure of driving risk. Additionally, it is possible that these databases are less likely to register crashes of lesser severity than are caregiver reports.

### Anosognosia as a determining factor for accidents

Anosognosia is generally defined as the lack of awareness of an illness. In the context of an individual with dementia, it means the loss of insight into one’s own cognitive or functional deficits. The frequency of anosognosia increases markedly with the severity of dementia, but is present in at least 10% of the patients with very mild dementia.^35^ Patients with anosognosia may engage in activities beyond their true capacity, thus being at high risk of exposure to potentially dangerous situations.

Starkstain et al. assessed 278 patients with AD and 45 age-comparable healthy controls using a comprehensive psychiatric and neuropsychological evaluation. Caregivers rated the frequency of patient’s commission of dangerous behaviors, including hazardous driving. The frequency of exposure to dangerous situations was 16% in the AD group and 2% in the control group. Anosognosia was the main clinical correlate of dangerous behavior, conferring a threefold increase in the risk of exposure to dangerous situations.^36^ Hunt et al. reported that 38% of the AD patients who failed a road test still considered themselves to be safe drivers.^37^ A number of studies have demonstrated the difficulty of drivers with dementia to recognize their deficits as well as their reluctance to relinquish driving privileges despite evidence of impaired capacity to drive safely.^29,38,39^

The association between anosognosia and frontal dysfunction has long been recognized.^40^ Recent studies using positron emission tomography have confirmed lower metabolism in the orbitofrontal structures of individuals with anosognosia.^41^ Accordingly, AD patients with deficits in frontal system function (e.g. disinhibition, inattention, judgment problems) or patients with frontotemporal dementia, may have a higher risk of continuing to drive beyond safe limits.

## Ethical and legal issues

Laws to protect the community in different countries are similar in considering that driving is not a right, but a privilege acknowledged by the state through issuing of a license. The role of health care professionals is not to determine whether any given individual should be granted a license, but they certainly are in a privileged position to identify patients who may pose a threat to themselves or others when driving.^42^

Limited judgment capacity and anosognosia may render demented individuals unable to make voluntary adjustments in their driving behavior. In such instances, the responsibility for driving cessation falls to individuals other than the patient. In a society where driving is a hallmark of independence, a recommendation to stop driving may be extremely hard to accept. In a struggle to maintain autonomy, the patient may offer resistance that the caregiver and the health care provider will have to confront.^43^

When a healthcare provider makes recommendations regarding driving capacity, conflicts may arise between the professional and the patient or family members. These issues can often be circumvented with thoughtful communication and attention to the individual patient’s needs. However, some patients or their families will not comply or agree with a physician’s recommendation to stop driving. In these cases physicians should ensure that the recommendation is passed on to someone with decision-making authority and should document this refusal in the medical records. This situation may also require a letter to the licensing agency. When disclosure is required, healthcare providers should seek to report the minimum amount of information necessary so as to respect patient confidentiality.^44^

Regulatory agencies in some countries have developed communication channels and legal processes to encourage reporting of hazardous driving. These mechanisms allow concerned physicians, police officers, family members, and others to report a driver for evaluation. Reporters are kept anonymous and protected from litigation, while the driver is invited to undergo a standardized evaluation in order to retain their license.^45^ In some countries, such as Brazil, public policies in this area are largely neglected and do not provide physicians with a uniform approach to assess driving fitness. An effort to develop clearly defined guidelines, establish communication channels, and improve the availability of on-road tests or driving simulators should reduce physicians’ dilemma and improve public safety.

## Driving risk assessment

### History taking

A report from the caregiver about driving performance should be obtained to specifically assess typical situations in which individuals with cognitive impairment have difficulties. These include lane change maneuvers, turning left at intersections, backing up, finding the way, reacting to unexpected maneuvers from other drivers, and reading traffic signs.^46^ The validity of the report should be considered, bearing in mind that caregivers may over- or underestimate the problems according to a number of clinical and psychosocial factors. Conducting this interview without the driver present may be necessary to avoid the possibility of the informant softening the reports. When the examining professional is faced with poor quality collateral information, a family member should be instructed to take responsibility for observing driving performance.

### Office-based tests

Although studies on older drivers have shown some evidence that office-based tests can be used to predict unsafe driving, these findings have not been validated in drivers with dementia. Molnar et al. conducted a systematic review of studies addressing in-office cognitive tests that could be used for assessing fitness to drive in persons with dementia. All the reviewed studies focused on association, without exploring cutoff scores or psychometric properties.^47^ From a clinical perspective, knowing that a test is associated with unsafe driving or motor vehicle crashes without information on cutoff scores is of little practical use. Healthcare professionals are faced with the difficult task of dichotomizing patients into safe versus unsafe to drive, a task that requires risk quantification.

In 2003, the American Medical Association (AMA) published a document entitled Physician’s Guide to Assessing and Counseling Older Drivers.^48^ The AMA guidelines recommend two office-based cognitive tests: the Trail Making Test, Part B (TMT-B) and the Clock Drawing Test (CDT). The TMT-B involves connecting numbers and letters randomly arranged on a page in alternating order. The score reflects the overall time required to complete the connections accurately. This test specifically assesses working memory, visuospatial skills, divided attention, and psychomotor coordination. There are numerous versions of the CDT, but they all involve asking the patient to draw the face of a clock, to put in all the numbers, and to set the time. The CDT assesses a patient’s long-term memory, short-term memory, visuospatial skills, selective attention, and executive skills. According to the AMA guidelines, a time for completion greater than 180 seconds on the TMT-B or any incorrect element on the CDT would signal a need for intervention. Although both TMT-B and CDT have been correlated to driving performance in studies with older adults, it is important to emphasize that these tests have not been validated prospectively in demented persons.

Some authors have suggested an empirical MMSE cut-off score, recommending that anyone who scores less than 24 should stop driving or undergo further assessment.^4,49^ However, in two prospective studies, the MMSE was not found to predict future crashes or violations among older individuals.^31,39^ In simulator and on-road studies, significant but modest correlations (range 0.4 to 0.6) were reported between MMSE and driving performance.^4^ The modest correlations observed probably reflect the fact that the MMSE may not be sensitive for mild impairments that affect driving competency. In addition, the test is weighted toward orientation, memory, and language, while lacks domains important to driving competence.

In conclusion, despite the large body of literature correlating cognitive and driving performance variables, there is insufficient evidence to employ any single test in frontline clinical settings. Clinicians should not apply patterns of cognitive assessment derived in a nondemented population without first confirming their predictive validity in patients with dementia.

### On-road testing

On-road testing protocols that are specific for dementia have been developed to obtain information about a patient’s actual driving behavior and to evaluate practical driving skills.^50^ The on-road tests are considered the preferred method when the information gathered from caregiver reports and cognitive testing are insufficient to reach a definitive conclusion. However, specialized on-road testing is not available in all locations, and where it is available, its substantial cost is usually borne by the patient. In places were standardized on-road tests are unavailable, adapted evaluations can be conducted by occupational therapists that may have specific training and experience in evaluating drivers with dementia.

## Strategies to achieve driving cessation

As soon as the diagnosis of dementia is made, clinicians should begin an open discussion anticipating competency issues. Information about impairments and its associated risks should be presented in an ongoing manner to patients and their families. Discussing driving cessation early in the disease allows clients and families to plan a smooth transition to a non-driving status while identifying ways to maintain their current lifestyle. Driving may be essential to maintain social activities and provide necessary transportation for self and spouse. Accordingly, driving cessation is a concrete representation of declining function that threatens the quality of life of demented patients.^51^

Caregivers are encouraged to gradually assume responsibility for providing transportation. Taxicabs, public transport or other alternatives should begin to be used early for increasing familiarity with transportation options. Gradually exposing these individuals to alternatives to the car is intended to decrease the risk of social isolation when driving cessation eventually occurs.^52^

A graduated program of driving reduction can be implemented when some degree of insight is preserved, by restricting driving to short distances, daylight hours, familiar routes and low traffic areas. Although there are insufficient data on the effectiveness of this strategy in reducing accidents, gradual restriction has been shown to be a practical option.

Some individuals will naturally adapt their driving behavior to interaction with a supervising co-pilot, which may help to avoid problems of getting lost, reading traffic signs, and even warning of dangerous situations. This strategy is not supported by the available evidence as effective in reducing accidents and should be used only when it is developed casually.^19^

If the physician recommends driving cessation, it is important to provide patients with objective reasons, preferably with the family members present. Patients may react better to arguments that emphasize the risk that they pose to others rather than to themselves. They may also respond to the argument that insurance companies will not pay for the damage caused by individuals who are driving against medical advice. Forced restraint from driving may be required for patients who do not comply with voluntary restriction. In these cases family members should be advised to limit the patient’s access to the vehicle by moving the car to a distant location, taking away the keys or disabling the vehicle.

## Conclusions

A significant proportion of individuals diagnosed with dementia are current drivers. When detecting cognitive impairment clinicians should routinely ask about driving status. Although visuospatial skills have been shown to correlate best with driving performance, there is not sufficient evidence to recommend any specific test for assessing driving abilities in individuals with dementia. Decision making regarding driving fitness should never be based on any single observation but rather on a set of data gathered from multiple sources.
